# Unraveling the Role of AtSRT2 in Energy Metabolism, Stress Responses, and Gene Expression during Osmotic Stress in *Arabidopsis thaliana*

**DOI:** 10.3390/plants13050711

**Published:** 2024-03-02

**Authors:** Alberto Obrecht, Manuel Paneque

**Affiliations:** 1 Doctoral Program in Biotechnology, Universidad de Santiago de Chile, Av. Lib. Bdo. O’Higgins 3363, Estación Central, Santiago 9170022, Chile; alberto.obrecht@usach.cl; 2 Department of Environmental Sciences and Natural Resources, Faculty of Agricultural Sciences, University of Chile, Santa Rosa 11.315, La Pintana, Santiago 8820808, Chile

**Keywords:** sirtuin, Sir2, osmotic stress, *Arabidopsis*, NAD+, mannitol, PEG

## Abstract

Sirtuins participate in chromatin remodeling and gene expression regulation during stress responses. They are the only deacetylases that couple the cellular NAD^+^-dependent energy metabolism with transcriptional regulation. They catalyze the production of nicotinamide, inhibiting sirtuin 2 (SIR2) activity in vivo. The SIR2 homolog, AtSRT2, deacetylates non-histone proteins associated with mitochondrial energy metabolism. To date, AtSRT2 mechanisms during stress responses in *Arabidopsis thaliana* remain unclear. The transduction of mitochondrial metabolic signals links the energy status to transcriptional regulation, growth, and stress responses. These signals induce changes by regulating nuclear gene expression. The present study aimed to determine the role of SRT2 and its product nicotinamide in the development of *A. thaliana* and the expression of osmotic stress-response genes. Leaf development was greater in *srt2+* plants than in the wild type, indicating that SET2 plays a role in energy metabolism. Treatment with polyethylene glycol activated and inhibited gene expression in *srt2-* and *srt2+* lines, respectively. Therefore, we concluded that SRT2-stimulated plant growth and repressed signaling are associated with osmotic stress.

## 1. Introduction

The silent information regulator 2 (SIR2) family of proteins (sirtuins) are NAD^+^-dependent deacetylases that lack structural or sequence homology with other deacetylases [1]. They can detect the energy status of the cell depending on the concentration of NAD^+^ and deacetylated proteins [2,3]. Their activity depends on the NAD^+^/NADH ratio and increases under conditions of nutritional or energy deficiency in mammals and yeasts [4,5].

Sirtuin-mediated deacetylation is coupled with NAD^+^ hydrolysis and the production of nicotinamide, which alters the NAD^+^ levels and triggers cellular changes [6,7,8,9]. Sirtuins were initially described as nuclear proteins with histone deacetylase activity that causes chromatin compaction and gene silencing. They have also been linked to increased longevity in yeast [10,11,12,13]. Recent studies have demonstrated that sirtuins are located in different organelles and deacetylate several non-histone proteins [14,15].

Sirtuins have been identified in bacteria (*Bacillus subtilis*) [16], archaea (*Sulfolobus solfataricus* L. major) [17], yeasts (*Saccharomyces cerevisiae*) [18], nematodes (*Caenorhabditis elegans*) [19], fruit flies (*Drosophila melanogaster*) [20], humans (*Homo sapiens*) [21], and plants (*Oryza sativa* and *Arabidopsis thaliana*) [22,23]. Protein deacetylation promotes changes in the enzymatic activity or function of target proteins [24,25]. Two SIR2 homologs, namely, sirtuin 1 (SRT1) and sirtuin 2 (SRT2), have been identified in *O. sativa* [22,26], *Vitis vinifera* [27,28], *Solanum lycopersicum* [29,30], and *A. thaliana* [23,31,32]. The functions of these enzymes have been studied in some organisms but not in plants [33].

To date, SRT1 has primarily been found in the nuclei of the studied plant species, whereas SRT2 is ubiquitous in different organelles [34]. SRT2 is localized in the mitochondria of *O. sativa* (OsSRT29) [22], mitochondria and chloroplasts of *V. vinifera* (VvSRT2) [28], and the nuclei and cytoplasm of *S. lycopersicum* (SiSRT2) [30]. SRT2 expression in grapevine is likely related to photosynthetic activity. Levels of VvSRT2 in the leaves correspond to the progression through developmental stages, where increased expression of VvSRT2 correlates with increased photosynthesis [28]. The rates of photosynthesis and VvSRT2 expression were found to be high during the inflorescence stage and gradually decreased over time [35]. However, the expression of VvSRT2 remained high in the leaves and was expressed in the flowers and fruits, but it was not expressed in the roots [36].

Little is known about the sirtuins in *A. thaliana* (ArSRT2) and other plant species [23,31,32]. AtSRT2 is localized in the nucleus [32] or mitochondria [23]. It negatively regulates biotic stress responses by silencing genes involved in salicylic acid signaling [32] and deacetylating non-histone proteins [23]. In addition, it physically interacts with various proteins, including ATP synthase and ATP/ADP transporters [23].

In AtSRT2 T-DNA insertion mutants, many proteins that interact with AtSRT2 are hyperacetylated [23]. Compared to that in wild-type (WT) plants, acetylation on the gamma and delta/epsilon subunits of ATP synthase was increased 1.9- and 2.1-fold, respectively; on subunit 8, it was increased 2.3-fold; and on subunit ATP17, it was increased 10.2-fold [23]. ATP17 is specific to plant ATP synthases. In addition, acetylation of ATP/ADP carrier proteins (AAC1 and AAC3) was increased 1.6-fold in AtSRT2 mutants compared to that in WT plants [23]. Changes in protein acetylation, linked to energy metabolism, did not result in any significant phenotypic changes. However, respiratory coupling to ATP synthesis was decreased, possibly through suppression of ATP synthase activity or an increase in mitochondrial decoupling in *srt2*-mutant plants [23].

SRT2-mediated deacetylation increases ATP synthase activity [23]. However, deacetylation of ATP/ADP transporters decreases its activity, as deacetylation increases ADP entry and decreases the ATP/ADP ratio in *srt2* mutants [23]. Disruption of SRT2 increases the concentration of amino acids, including glutamine and glycine, and decreases the concentration of important metabolites, including sugars (fructose, glucose, erythritol, and myoinositol), amino acids (serine, proline, arginine, threonine, tyrosine, and alanine), and organic acids (shikimic, ascorbic, pyruvic, fumaric, and gamma-aminobutyric acids) [23]. Furthermore, SRT2 regulates the energy metabolism in cells, inducing changes in metabolites that may serve as signaling molecules. This activates or represses the downstream pathways, allowing plants to adapt to their current energy status.

Although AtSRT2 is associated with the regulation of energy metabolism [23], its role in the stress response of *A. thaliana* has not been fully elucidated. Mitochondrial metabolic signals can induce changes in other organelles [37], including the nucleus, by regulating nuclear gene expression. These signals are transduced by proteins such as the target of rapamycin (TOR) and SNF1-related kinases (SnRK1), which couple the energy and nutritional status of the cell to transcriptional regulation, growth, and stress responses [38,39].

The objective of the present study was to evaluate the role of SRT2 during the development and osmotic stress response in *A. thaliana* through phenotypic analyses. In *A. thaliana*, SRT2 is located in the mitochondria [23]. Therefore, in this study, we observed the phenotypic changes in plant development and the transcription of stress-response genes related to SRT2-dependent changes in mitochondrial metabolism. Moreover, we investigated the effect of *srt2* overexpression and silencing on plant development and stress-related gene expression.

## 2. Results and Discussion

### 2.1. Effect of SRT2 on the Development of A. thaliana

Phenotypic changes caused by SRT2 deletion or overexpression during the development of *A. thaliana* were evaluated by examining the morphology of the seeds, roots, leaves, and siliques.

**Figure 1:**
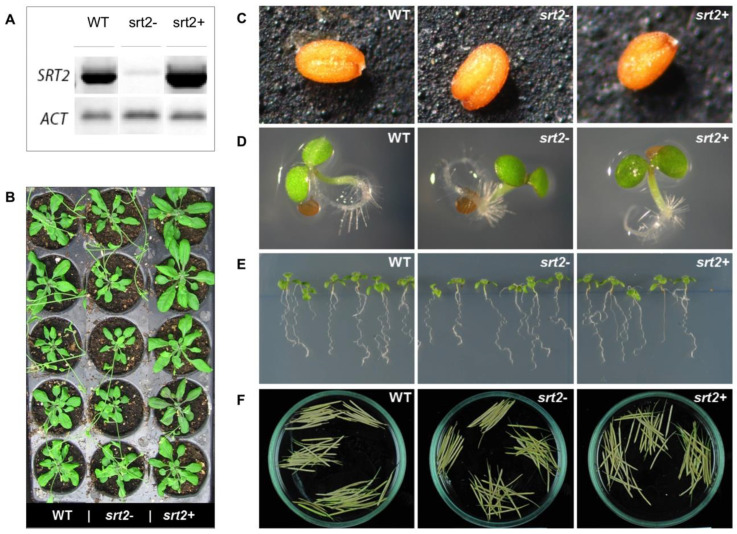
*Srt2* affects the leaf size in Arabidopsis thaliana, as shown by a qualitative, phenotypic analysis of *A. thaliana* genetic lines, *srt2*+, *srt2*-, and wild type (WT). (**A**) Semi-quantitative RT-PCR of the *srt2*- deletion line and *srt2*+ overexpression line. (**B**) Plants grown in vermiculite–peat soils for 23 long days. (**C**) Seed morphology and size. (**D**) Development of 3-day-old seedlings cultivated in MS medium. (**E**) Root development in 16-day-old seedlings cultivated in vertical plates in MS medium. (**F**) Silique morphology and size.

Gene expression in the *srt2+*, *srt2-*, and WT lines was analyzed using reverse-transcription polymerase chain reaction (RT-PCR). The deletion line (*srt2-*) exhibited minimal SRT2 expression, whereas the overexpression line (*srt2+*) showed higher expression levels than the WT line (Figure 1A). The *srt2-* plants had noticeably shorter and thinner leaves compared to those of the *srt2*+ and WT plants (Figure 1B). However, there were no observed abnormalities in the development of seeds (Figure 1C), seedlings (Figure 1D), roots (Figure 1E), or silique morphology (Figure 1F) among the three genetic lines.

The *srt2+* plants had larger leaves and were more vigorous than *srt2-* and WT plants (Figure 1B). These differences were greater in plants grown during longer days. In contrast, the two mutant lines developed normally, with no significant differences in seed characteristics, seedling morphology, root development, or silique morphology. The length of the siliques, number of seeds per silique, number of siliques per plant, and seed width, length, and area were measured (Table 1). Compared to the WT plants, the *srt2+* and *srt2-* plants had shorter siliques, fewer seeds per silique, and fewer siliques per plant. These differences were discrete and did not decrease the germination percentage or seedling viability.

We observed that neither SRT2 deletion nor its overexpression led to major changes in the morphology of seeds and siliques during the development of *A. thaliana*. The siliques were shorter in the mutant plants than in the WT plants (Table 1). The reduction in silique length was greater in *srt2+* plants than in *srt2*- and WT plants. In addition, the number of seeds per silique was reduced in *srt2+* and *srt2-* plants (Table 1), and the reduction was greater in *srt2-* than in *srt2+*. The number of siliques per plant did not significantly differ among the three genetic lines (Table 1).

In comparison with those of the WT plants, the length of the siliques and the number of seeds per silique were slightly lower in the mutant plants. However, we could not infer whether SRT2 directly affected the growth of siliques or seed formation. Possible changes in energy metabolism caused by SRT2 deletion or overexpression were more pronounced in vegetative tissues, such as leaves, than in reproductive tissues.

In comparison with that in WT plants, the silique length in the mutant plants was decreased, and the reduction was more pronounced in *srt2+* plants than in *srt2-* plants (Table 1). In addition, the number of seeds per silique was lower in the mutant plants than in the WT plants, and the reduction in seed number was greater in the *srt2-* plants than in *srt2+* plants. No significant differences were observed in the number of siliques per plant among the three lines. Previous reports have indicated that the inhibition of SRT2 enhances auxin production [40], and although auxins are crucial phytohormones for plant growth optimization [41], excessive levels can inhibit growth once optimal concentrations are exceeded [42]. Therefore, it is hypothesized that SRT inhibition could lead to unregulated auxin production or transport, subsequently inhibiting growth. Conversely, if SRT2 is implicated in auxin production or transport [10,40], its overexpression might reduce or inhibit auxin production or transport, thereby hindering growth if auxin levels fall below optimal concentrations.

**Figure 2:**
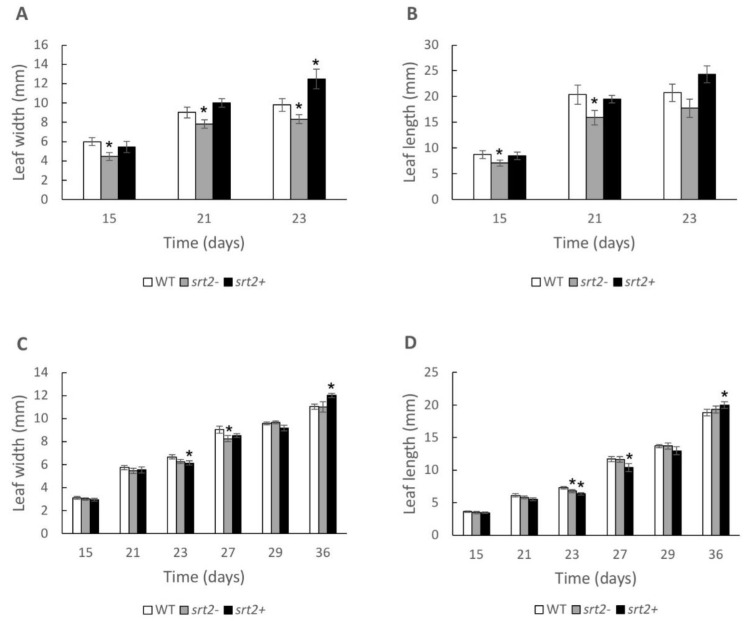
*Srt2* affects the leaf length and width in *Arabidopsis thaliana* grown in long- and short-day photoperiods. The leaves of *srt2*+, *srt2*-, and WT genetic lines of *A. thaliana* grown in vermiculite-peat soil (3:1) were measured on days 15, 21, and 23 for the long-day photoperiod conditions and on days 15, 21, 23, 27, 29, and 36 for the short-day photoperiod conditions. (**A**) Width of the leaves in the long-day photoperiod. (**B**) Length of the leaves during the long-day photoperiod. (**C**) Width of the leaves during the short-day photoperiod. (**D**) Length of the leaves during the short-day photoperiod (n = 9; two leaves per plant and nine plants per row). The asterisks indicate significant differences compared with the WT control, as calculated using the Mann–Whitney U test (*p* < 0.05).

Leaf growth, as measured by width and length, was higher in the *srt2+* plants than in the WT plants on the last day of measurement, corresponding to maximum leaf development (Figure 2A,C,D). In contrast, leaf growth was lower in the *srt2-* line than in the WT line (Figure 2A,B).

Leaf growth was higher in the *srt2+* plants cultivated for 23 long days (16 h days) than in the WT plants (Figure 2A). Leaf development in plants cultivated under a short photoperiod (8 h days) was similar in the three genetic lines. However, *srt2+* plants still had wider leaves than those of the WT plants on day 36 (Figure 2C). The most evident differences were observed on the final day of measurement (Figure 2A–C). Longer *srt2+* leaves were slightly thinner (Figure 2C), but still slightly shorter (Figure 2D), than those of the WT plants before maximum leaf development. This observation was reversed starting on day 29.

Plant growth was the highest in the *srt2+* plants, particularly during the long-day photoperiod (Figure 1A). This suggests that SRT2 may be responsible for increasing energy-utilization efficiency. Sirtuins in yeasts and mammals affect several processes depending on their subcellular locations [34,43]. SRT2 is exclusively located in the mitochondria and binds to the mitochondrial proteins involved in energy metabolism, including ATP synthase and ATP/ADP transporters [23].

SRT2 modulates protein activity via lysine deacetylation. In the present study, the coupling of mitochondrial respiration and ATP synthesis was reduced in the *srt2-* plants compared to that in the *srt2*+ plants. Moreover, SRT2 deletion is known to decrease glucose levels and change the concentrations of other metabolites, including amino acids, organic acids, and sugars [23]. Therefore, SRT2 can promote changes in the plant’s energy status, which can, in turn, trigger changes in protein synthesis/degradation and gene expression/repression via the activation or repression of the TOR pathway and its antagonist SnRK1. TOR and SnRK1 kinases can sense the energy status of the cell and modulate protein transcription and synthesis to adapt to a new energy state, resulting in either growth or autophagy [38,44].

The observed phenotypic differences among the three genetic lines can be attributed to differences in mitochondrial coupling or ATP synthase activity, both of which are affected by SRT2 activity. SRT2 overexpression may have increased several parameters, including plant photosynthetic efficiency, the ATP/ADP ratio, and glucose levels. Increased glucose levels can activate the TOR signaling pathway, which is mediated by Glc-TOR [44]. Together with an increase in energy efficiency, the activation of TOR can explain the increase in plant vigor and leaf growth in the *srt2+* line. Therefore, TOR may be an effector of SRT2 activity in the mitochondria.

### 2.2. Root Length under Stress Conditions

**Figure 3:**
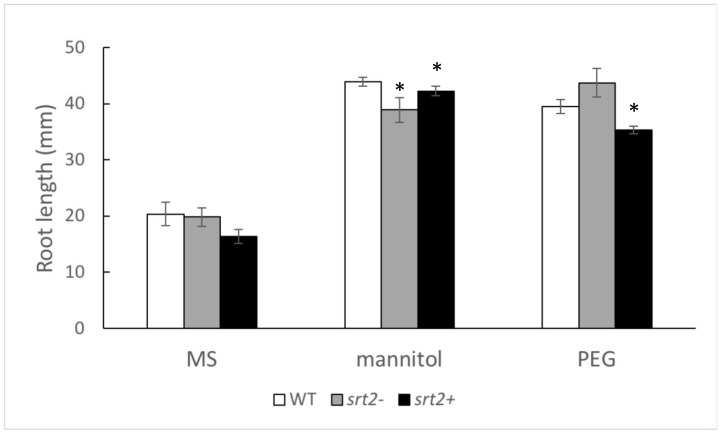
Osmotic stress affects the root length of the three genetic lines of *Arabidopsis thaliana* plants grown under osmotic stress. The root length of *A. thaliana* seedlings grown on vertical plates was measured after 16 days of treatment with the control MS medium, 50 mM of mannitol, or 2.5% polyethylene glycol (PEG). The asterisks indicate significant differences compared to the WT control, as calculated using the Mann–Whitney U test (*p* < 0.05).

Treatment with 50 mM of mannitol and 2.5% polyethylene glycol (PEG) increased the root length compared to that of the control (grown in only MS medium) at 16 days post-germination (Figure 3). Under mannitol treatment, both *srt2*- and *srt2*+ lines exhibited shorter roots compared to the WT, whereas only the *srt2*+ line had shorter roots compared to WT when treated with PEG (Figure 3).

All three lines responded similarly to osmotic stress, both for mannitol and PEG, by extending their roots into the culture medium. Differences in root growth among the three plant lines were discrete. According to König et al. (2014) [23], SRT2 does not directly influence plant phenotype but plays a role in metabolite transport. Consequently, its mechanisms of action may not manifest in noticeable morphological changes.

### 2.3. Membrane Lipoperoxidation

**Figure 4:**
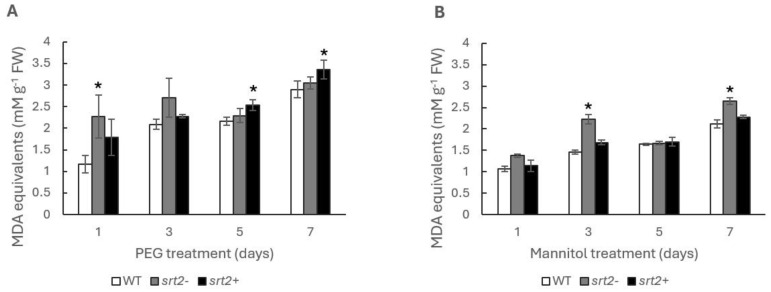
Membrane lipoperoxidation in plants subjected to two types of osmotic stress. The malondialdehyde (MDA) level per gram of fresh weight was determined after 1, 3, 5, and 7 days of treatment with (**A**) 30% polyethylene glycol (PEG) or (**B**) 300 mM of mannitol in the *srt2*+, *srt2*-, and WT lines of *Arabidopsis thaliana* cultivated in a liquid medium for 10 days. The asterisks indicate significant differences compared with the WT control, as calculated using the Mann–Whitney U test (*p* < 0.05) (n = 3).

The plants were grown in multi-well plates and subjected to osmotic stress by treatment with 300 mM of mannitol and 30% PEG. This methodology allowed multiple assays to be performed simultaneously under the same experimental conditions. Ten seeds were allowed to germinate per well. After 10 days of germination, stress-induced production of malondialdehyde (MDA) was quantified. The MDA levels in the *srt2+* plants were higher than those in the WT plants. The rate of lipoperoxidation was higher in the *srt2-* plants grown in MS medium only and after 3 and 7 days of incubation with mannitol than in WT plants (Figure 4A,B).

The increase in MDA levels was a function of the duration of stressor exposure in all three genetic lines. No significant differences in MDA levels were observed among the three genetic lines after the different treatments. The putative increase in energy efficiency in the *srt2+* line did not increase the neutralization of membrane lipoperoxides induced by the treatments. This may be because the experiments were conducted on seedlings during the first week of growth, and in this early stage plant, the morphology among three genetic lines was similar. However, phenotypic differences in response to osmotic stress are possible in adult plants, which may be the result of an increase in the stress response in *srt2+* plants.

### 2.4. SRT2 Is Critical for Activating Osmotic Stress Response Genes

**Figure 5:**
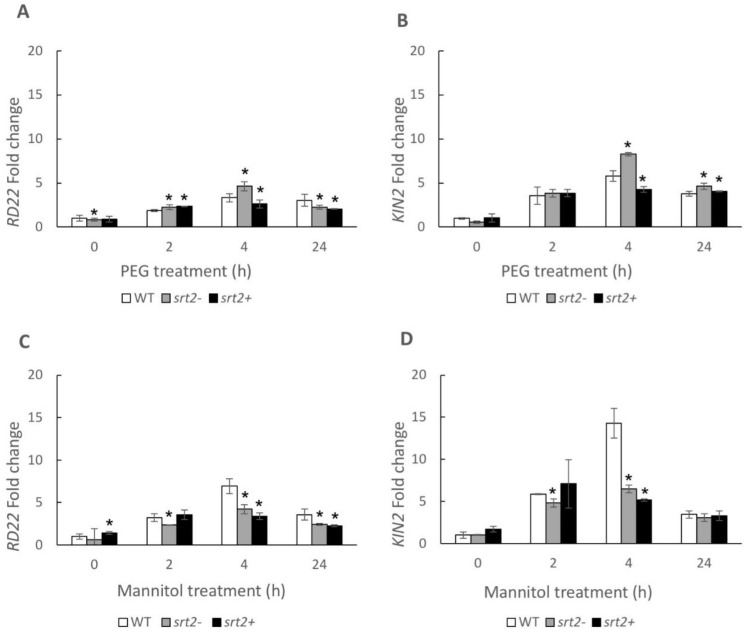
Short-term changes in the expression of osmotic stress marker genes (*kin2* and *rd22*) in plants subjected to two types of osmotic stress. Changes in their expression in the *srt2*+, *srt2*-, and WT control plants of *Arabidopsis thaliana* grown in a liquid medium for 10 days were determined after 0, 2, 4, and 24 h of treatment. The plants were grown in (**A**,**B**) MS medium + 30% polyethylene glycol (PEG), or (**C**,**D**) MS medium + 300 mM of mannitol. *Actin2* was used as a housekeeping gene. The results are relative to the control WT expression at t = 0 h. The asterisks indicate significant differences compared with the WT control, as calculated using the Mann–Whitney U test (*p* < 0.05).

Treatment with 300 mM of mannitol increased the expression of the marker genes *kin2* and *rd22* in the WT plants. An increase in *kin2* and *rd22* expression was expected, as these genes are markers of osmotic stress. After the treatment with mannitol for 4 h, the expression levels of *kin2* and *rd22* were significantly lower in the mutant plants than in the WT plants (Figure 5).

After 4 h of incubation with 30% PEG, *kin2* and *rd22* expression increased in *srt2-* and WT plants but decreased in *srt2+* plants. The expression of these genes in response to osmotic stress was significantly different among the groups evaluated and was more pronounced after 4 h of incubation with 30% PEG. Therefore, SRT2 indirectly modulates the expression of these genes, and this activity is part of the stress-signaling pathway in *A. thaliana*. The post-treatment expression profiles of both genes were similar (Figure 5).

This study aimed to assess the role of SRT2 in the activation or repression of stress marker genes in response to osmotic stress caused by these two stressors. After incubation with PEG for 4 h, the expression of these genes was suppressed in the *srt2+* line but activated in the *srt2-* line. In contrast, mannitol treatment suppressed their expression in both mutant lines. These results indicated that gene repression was independent of SRT2 expression.

Therefore, SRT2 modulated gene expression by indirectly silencing *kin2* and *rd22.* This activity is part of the stress signaling pathway in response to PEG treatment in *A. thaliana*. However, mannitol did not have the same effect on gene expression. This may be because mannitol can penetrate the apoplast and is taken up by cells [45], attenuating the effective osmotic gradient between the medium and the symplast. Another reason may be that the hydric potential generated by mannitol (−0.74 MPa) is much lower than that generated by PEG (−10.27 MPa).

### 2.5. Nicotinamide’s Contribution to Stress Signaling

**Figure 6:**
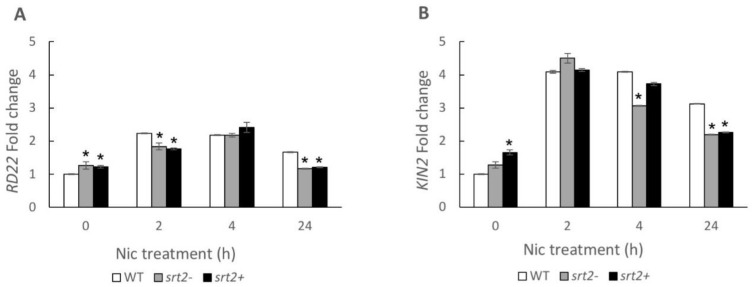
Short-term changes in the expression of osmotic stress marker genes (*kin2* and *rd22*) in plants treated with 2.5 mM of nicotinamide. The seedlings of *srt2*+, *srt2*-, and WT control lines of *Arabidopsis thaliana* were grown in a liquid medium for 10 days and treated with 2.5 mM of nicotinamide. The fold changes in the osmotic stress marker genes, (**A**) *kin2* and (**B**) *rd22*, were determined after 0, 2, 4, and 24 h of treatment with nicotinamide. Actin2 was used as a housekeeping gene. The results are presented relative to the control WT expression at t = 0 h. The asterisks indicate significant differences compared with the WT control using the Mann–Whitney U test (*p* < 0.05) (n = 3).

SRT2-mediated signaling during the osmotic stress response may be nicotinamide-dependent. The differences in the expression of osmotic stress marker genes induced by nicotinamide were measured. In the presence of 2.5 mM of nicotinamide, the expression of *rd22* was slightly increased in all three genetic lines, and the expression was the highest after 4 h of incubation (Figure 6). After 2 and 4 h of treatment, *kin2* was activated in all three genetic lines. However, its expression in the *srt2+* and *srt2-* plants declined rapidly beyond 4 h after the treatment.

**Figure 7:**
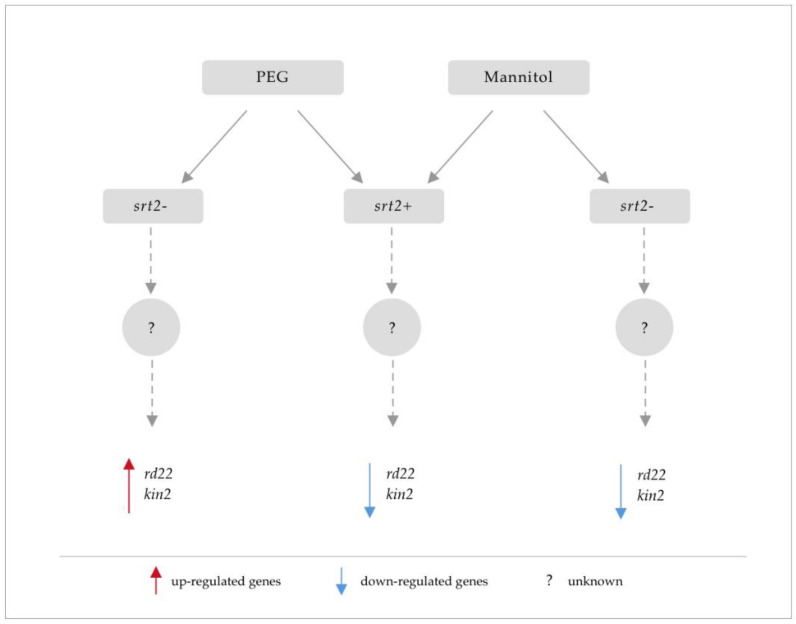
Proposed mechanism of SRT2 activity in the sirtuin-dependent, nicotinamide-independent defense response. Diagrammatic representation of osmotic stress-dependent genes with sensitivity to mannitol and polyethylene glycol (PEG) in the *srt2* mutants compared with those in the wild type (WT).

Nicotinamide did not induce SRT2-mediated osmotic stress signaling (Figure 6). This finding indicated that it did not directly participate in the activation of SRT2-mediated stress signaling in *A. thaliana*. The SRT2-dependent, nicotinamide-independent defense response model proposed for *A. thaliana* is depicted in Figure 7.

Mitochondrial SRT2 produces signals associated with the regulation of stress marker gene expression. These signals, including changes in mitochondrial coupling, energy efficiency, and increased glucose levels, can be sensed and transmitted to the nucleus to modulate gene expression and increase the plant’s ability to adapt to a new state of energy or stress. SnRKs and TOR promote the coupling of these retrograde signals, as these molecules are synthesized in the mitochondria and affect the nuclear processes, such as transcription.

**Figure 8:**
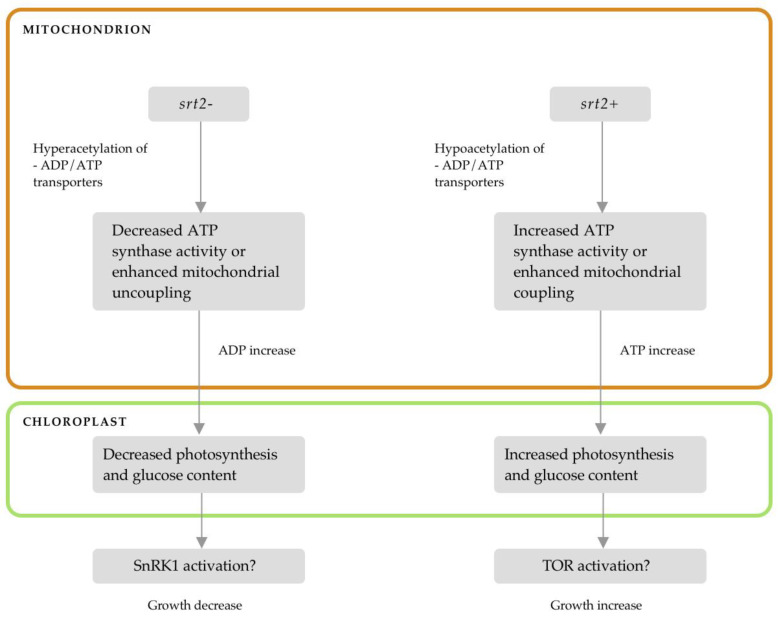
Possible mechanism underlying the variations in leaf growth. Disruption of SRT2 causes hyperacetylation of critical mitochondrial proteins, resulting in a decrease in ATP synthase activity and an increase in the concentration of photosynthetic products such as glucose. This model proposes that SRT2 overexpression has the opposite effect, reducing mitochondrial protein acetylation and increasing ATP synthase activity and glucose levels. The putative increase in glucose levels may be responsible for the increased leaf growth in *srt2*+ plants, which is mediated by TOR.

In the mitochondria, SRT2 binds to and deacetylates critical proteins related to cellular respiration, including ATP/ADP transporters, AAC1, and AAC3, as well as ATP synthase. Proteins in the *srt2+* plants were hyperacetylated, which led to decreased ATP synthase activity, mitochondrial coupling, photosynthetic rate, and concentration of sugars such as glucose (Figure 8).

Low energy levels can activate SnRK1 and trigger autophagy. This may have been the cause of the reduced leaf development in *srt2-* plants. In contrast, these mitochondrial proteins may be hypoacetylated in *srt2+* plants, resulting in increased ATP synthase activity or mitochondrial coupling. This could result in a higher energy state in which SnRK1 is inhibited and TOR is activated, explaining the increased leaf growth (Figure 8).

TOR is involved in osmotic stress signaling [46] and promotes root growth by modulating gene expression. Glc-TOR signaling also involves several chromatin modulators, transcription factors, signaling regulators, and growth and stress response proteins [44]. Therefore, the activation of TOR affects nuclear gene expression and may be partially responsible for the changes in gene expression observed in this study.

## 3. Materials and Methods

### 3.1. Plant Material, Growth Conditions, and Stress Treatment

*A. thaliana* ecotype Columbia (Col) was used as the WT control. The *srt2* mutants (Salk_149295) were obtained from ABRC (http://www.arabidopsis.org/abrc (accessed on 15 august 2003), and homozygous mutant lines were identified according to the Salk protocol (http://signal.salk.edu/tdnaprimers.2.html (accessed on 28 July 2005). Plants homozygous for T-DNA insertion were confirmed by PCR amplification using primers corresponding to sequences flanking the T-DNA insertion site and gene-specific primers. The primers used to identify the homozygous plants are listed in Table 2. Transgenic plants overexpressing AtSRT2 (OE) were generated in the Col-0 background. The AtSRT2-CDS3 sequence was amplified from the cDNA of WT (Col-0) plants using high-fidelity DNA polymerase KOD-plus (Toyobo, Osaka, Japan). The forward and reverse primer sequences are listed in Table 3. The amplified DNA was inserted into the pENTR/D-TOPO cloning vector (Invitrogen, Taastrup, Denmark), followed by site-directed recombination to generate the binary vectors pGWB2 and pGWB5 for SRT2-OE and 35S:SRT2-GFP, respectively [47]. The binary pGWB5-AtSRT2 plasmid was transformed into *Agrobacterium tumefaciens* strain GV3101 (pMP90). Transformation of *A. thaliana* was performed using the floral dip method [48]. To screen the transformants, seeds were grown on plates containing Murashige and Skoog (MS) medium supplemented with 40 μg/mL of hygromycin B (Roche Diagnostics, Mannheim, Germany). The resistant plants were then transferred to the soil for further analysis. The seeds were surface-sterilized and sown on the MS agar plates containing full-strength MS salts, 0.8% (*w*/*v*) agar, and 1% (*w*/*v*) sucrose. They were stratified in the dark at 4 °C for four days and then either transferred to a growth chamber with a 16 h/8 h light (350 μmol m^−2^s^−1^)/dark cycle at 23 °C or directly sown in the soil after stratification under the same conditions.

*A. thaliana* seeds were surface-sterilized and sown in 24-well tissue culture clusters at 20 seeds per well (Costar Corp., Cambridge, MA, USA). Each well contained 1 mL of sterile MS medium (MS salts; ICN Hubber, Barcelona, Spain) supplemented with 0.5% sucrose. The seeds were grown with shaking (150 rpm) in a culture room under a 16 h day (26 °C)/8 h night (22 °C) diurnal cycle. Fresh medium (500 µL) was added to each well eight days after sowing, and the experiments were conducted after two days. The medium remaining in the wells was removed prior to initiating the treatment, and 1 mL of fresh medium was added to each well. The test compounds were diluted directly in the wells to their final concentrations from the stock solutions, which were prepared by separately mixing 300 mM of D-mannitol and polyethylene glycol (PEG; molecular weight of 6000 kDa) from Sigma-Aldrich (St. Louis, MO, USA), with water to obtain a 30% (*w*/*w*) aqueous solution. To study the germination of plants grown on the agar plates, the surface-sterilized seeds were planted in 1/2 MS medium supplemented with 0.8% phytagel, cold-treated for two days, and grown under the same conditions as the soil-grown plants. This medium allows solid culture, long root production, and easy root cleaning [49,50]. The plants were sown onto a mesh and cultured for 16 days before careful extraction and washing with sterile water. All experiments were performed independently at least three times, yielding highly reproducible results.

### 3.2. Greenhouse Growth Conditions

The seeds were stratified in the dark at 4 °C for 4 days and then either transferred to a growth chamber with 16 h/8 h light or 8 h/16 h light (350 μmol m^−2^s^−1^)/dark cycle at 23 °C or directly sown in the soil after stratification under the same conditions. A nutritive solution (pH 6.5) comprising the following macro and micronutrients was applied to the plants: 4 mM of KNO_3_, 4 mM of Ca(NO_3_)_2_, 1.5 mM of MgSO_4_, 0.75 mM of KH_2_PO_4_, 0.035% (p/v) EDTA-FeSO_4_, 70 μM of H_3_BO_3_, 14 μM of MnCl_2_, 0.5 μM of CuSO_4_, 1 μM of ZnSO_4_, 0.2 μM of Na_2_MoO_4_, 10 μM of NaCl, and 0.01 μM of CoCl_2_.

### 3.3. Measurement of Leaf Properties

The dimensions of the plant leaves grown on long and short days were measured. Leaf length and width were measured using the AxioVision Rel. Software (version 4.6). The number of siliques growing on the main stem, their length, and number of seeds per silique were measured for each plant. The length of the mature siliques of plants grown in the soil was determined by capturing the images of the siliques and analyzing them later using EZ-Rhizo software (version 1.0.0.0). Three technical replicates were designed for each genetic line, and the median and standard error were calculated for each.

### 3.4. Germination Assay

Germination experiments were performed using fully ripened seeds of *A. thaliana*. The seeds were surface-sterilized and plated in groups of 50–100 in each plastic Petri dish containing MS medium without hormones or vitamins [51] supplemented with 2% (*w*/*v*) sucrose and 0.7% (*w*/*v*) agar and adjusted to pH 7.0. The seeds were incubated for 24 h at 4 °C and subsequently grown in a growth chamber at 24 °C under continuous white light (8.4 μmol s^−1^m^−2^) for 50 h. Ruptures of the testa and endosperm were scored by observation using a binocular microscope [52]. Four replicates were used for each treatment group. The seeds were photographed using a Canon G10 digital camera (Bensheim, Germany) attached to a stereomicroscope (Stemi 2000 C; Carl Zeiss, Oberkochen, Germany). AxioVision Rel. software (version 4.6) and Adobe Photoshop CS3 were used to analyze the images.

### 3.5. Lipid Peroxidation

Malondialdehyde (MDA) assay was conducted to estimate the level of lipid peroxidation in the leaf tissues following the method described by Hodges et al. [53] with minor modifications. Briefly, the leaf discs (0.1 g) were ground, suspended in 2 mL of ice-cold 1% *w*/*v* trichloroacetic acid (TCA), and centrifuged at 10,000× *g* for 5 min. Subsequently, 0.25 mL of the supernatant was mixed with 1 mL of 0.5% *w*/*v* thiobarbituric acid (TBA), incubated at 100 °C for 30 min, and immediately chilled. Differences in absorbance at 532 and 600 nm were measured using a Shimadzu UV-1601 spectrophotometer (Tokyo, Japan). An extinction coefficient of 155 mM^−1^ cm^−1^ was used for MDA determination.

### 3.6. RNA Extraction and First Strand cDNA Synthesis

The sampled plants were frozen in liquid nitrogen and ground to a fine powder using a mortar and pestle; 100 mg of this material was used for RNA isolation. Total RNA was extracted using the TRIzol reagent (TIANGEN, Beijing, China) in accordance with the manufacturer’s instructions. The isolated RNA was treated with RNase-free DNase (Takara Bio, Kusatsu, Japan) to eliminate genomic DNA contamination. The purity and concentration of the RNA samples were measured with a Q5000 micro-volume UV-Vis spectrophotometer (Quawell Technology, San Jose, CA, USA). The integrity of the samples was examined by agarose gel electrophoresis. RNA samples with 260/280 nm absorbencies between 1.9 and 2.1 were used for subsequent experiments. First-strand cDNA synthesis was performed using a cDNA synthesis kit (Sangon, Shanghai, China) in accordance with the manufacturer’s instructions, with a total volume of 20 μL containing 2 μg of total RNA. RNA extraction and cDNA synthesis were performed in triplicate for each sample. The cDNA solution was diluted 10 times with nuclease-free water, and the aliquots were stored at −20 °C until further use in RT-qPCR [54,55].

### 3.7. qRT-PCR Analysis

qRT-PCR analysis was performed in 96-well plates on an Mx3005 multiplex system (Stratagene, La Jolla, CA, USA) using EvaGreen-based PCR assay. Each reaction mixture had a final volume of 20 µL containing 1.0 μL of the template, 10.0 μL of 2× SuperReal Premix Plus (SYBR Green, Tiangen Biotech, Beijing, China), 0.4 μL of 50× ROX Reference Dye, 0.6 μL of each primer (10 μmol/μL), and 6.2 μL of double-distilled water. The reaction steps were as follows: pre-denaturation at 94 °C for 40 s, followed by 38 cycles of denaturation at 94 °C for 10 s and annealing at 57.2 °C for 30 s, and a final extension step at 72 °C for 10 min [54]. Three technical replicates were used for each cDNA sample.

### 3.8. Molecular Markers of Abiotic Stress Response

The expressions of molecular markers of abiotic stress response, including *kin2* (At5g15970) [56,57,58,59,60,61], *rd22* (At5g25610) [59,62,63,64,65,66], and the constitutive expression of *ACT2* (At3g18780), which is commonly used as a reference gene for qRT-PCR, were studied. Table 4 shows the primer sequences used, amplicon length in base pairs (bp), and melting temperature (Tm).

### 3.9. Statistical Analyses

All the graphs were prepared in Microsoft Excel. Statistical analyses were carried out using GRAPHPAD PRISM 8 software. The Mann–Whitney U test was used to obtain significant differences compared with the WT (*p* < 0.05 was considered statistically significant).

## 4. Conclusions

In *Arabidopsis thaliana*, the sirtuin SRT2 is a mitochondrial deacetylase that binds to and deacetylates proteins involved in the electron transport chain. The results of this study indicated that SRT2 was not a major participant in plant development but regulated energy efficiency by modulating its cellular energy status and/or leaf growth under normal growth conditions. This was demonstrated in the *srt2+* overexpressing line, which exhibited greater leaf growth than that of the WT control. Moreover, our results indicated that SRT2 may be involved in signaling under conditions of osmotic stress generated by PEG or mannitol, as the deletion or overexpression of SRT2 induced changes in the expression of stress marker genes. However, SRT2 did not affect plant resistance to osmotic stress in the first developmental stages, as evidenced by no significant intergroup differences in MDA levels, initial seedling growth, or root growth.

## Figures and Tables

**Table 1:** *Srt2* affects the silique length and number of seeds per silique in *Arabidopsis thaliana*. The parameters of interest were analyzed in the *srt2+*, *srt2*-, and WT lines; these included the length of the mature siliques (60 siliques per row); number of seeds per silique (60 siliques per row); number of siliques per plant (24 plants per row); and seed width, length, and area as an average of four seeds. All plants evaluated in this study were cultivated in soil.

Genotype	WT	*srt2-*	*srt2+*
Feature	Mean ± SD	Mean ± SD	Mean ± SD
Silique length (mm)	13.93 ± 0.16	13.53 ± 0.12 *	13.01 ± 0.12 *
Seed number per silique	48.67 ± 0.95	44.49 ± 0.82 *	46.63 ± 0.89
Siliques number per plant	90.21 ± 4.21	82.41 ± 4.92	87.042 ± 4.03
Seed width (mm)	0.40 ± 0.03	0.45 ± 0.02	0.44 ± 0.02
Seed length (mm)	0.72 ± 0.03	0.75 ± 0.01	0.73 ± 0.03
Seed area (mm^2^)	0.23 ± 0.04	0.26 ± 0.03	0.25 ± 0.02

*: Significant differences in silique length and number of seeds per silique compared to those of the WT control, as calculated using the Mann–Whitney U test (*p* < 0.05).

**Table 2:** Primers used for the identification of homozygous plants.

Oligonucleotide	Sequence
SALK_N649295-PCR1-D	5′-CGC AGA GAG AGA ACA AAA TCG-3′
SALK_N649295-PCR1-R	5′-TTC CAC ATT CTG TGC TAA CCC-3′
SALK_N649295-PCR2-D	5′-TGG TTC ACG TAG TGG GCC ATC G-3′
SALK_N649295-PCR2-D	5′-TTC CAC ATT CTG TGC TAA CCC-3′
SALK_N58811-PCR1-D	5′-AGA GTT CAT AAA AAC AAT GAA TCA AG-3′
SALK_N58811-PCR1-R	5′-AAC TCA TTG CAT TTG CAT AGG-3′
SALK_N58811-PCR2-D	5′-AAC TCA TTG CAT TTG CAT AGG-3′
SALK_N58811-PCR2-R	5′-TGG TTC ACG TAG TGG GCC ATC G-3′
SALK_N631994-PCR1-D	5′-TTT AAG GCA TTT TCA AGG CTG-3′
SALK_N631994-PCR1-R	5′-GGA GTT TAC TCG ATC AAG CCG-3′
SALK_N631994-PCR1-D	5′-GGA GTT TAC TCG ATC AAG CCG-3′
SALK_N631994-PCR1-R	5′-TGG TTC ACG TAG TGG GCC ATC G-3′

**Table 3:** Primers used to amplify the CDS3 of AtSRT2.

Oligonucleotide	Sequence
AtSRT2-CDS3-LP	5′-AGAAGCCGCACTCTAAGCAC-3′
AtSRT2-CDS3-RP	5′-AGTTACGCTGGATGGAGGAG-3′

**Table 4:** Primers for reference genes and *srt2* for RT-qPCR.

Gene	Gene Code	Primer Sequence (Forward/Reverse)	Amplicon Length (bp)	Tm °C
Act2	At3g18780	5′-TGCCAATCTACGAGGGTTTC-3′	203	57.3
		5′-TGAGGTTTCCATCTCCTGCT-3′		57.3
Kin2-LP	At5g15970	5′-ACCAACAAGAATGCCTTCCA-3′	146	55.2
		5′-ACTGCCGCATCCGATATACT-3′		57.3
RD22-LP	At5g25610	5′-GGGCTGTTTCCACTGAGGT-3′	160	58.8
		5′-GTCGTCATCATCGCCTTGT-3′		56.7
SRT2-LP	At5g09230	5′-AGTTACGCTGGATGGAGGAG-3′	177	59.4
		5′-AGAAGCCGCACTCTAAGCAC-3′		59.4

## Data Availability

Data are contained within the article.
